# Energy Efficiency in RF Energy Harvesting-Powered Distributed Antenna Systems for the Internet of Things

**DOI:** 10.3390/s20164631

**Published:** 2020-08-18

**Authors:** Jiaxin Li, Ke Xiong, Jie Cao, Xi Yang, Tong Liu

**Affiliations:** 1 School of Computer and Information Technology, Beijing Jiaotong University, Beijing 100044, China; ll_jiaxin@163.com (J.L.); kxiong@bjtu.edu.cn (K.X.); 2 School of Computer and Communication Engineering, University of Science and Technology Beijing, Beijing 100083, China; 3 Beijing Key Laboratory of Traffic Data Analysis and Mining, Beijing Jiaotong University, Beijing 100044, China; 4 College of Information Engineering, Nanjing University of Finance & Economic, Nanjing 210023, China; Jie.Cao@nufe.edu.cn; 5 School of Information, Beijing Wuzi University, Beijing 101149, China; 6 Beijing Intelligent Logistics System Collaborative Innovation Center, Beijing 101149, China; 7 Beijing Computing Center, Beike Industry Park, Beijing 100094, China; Liutong@bcc.ac.cn

**Keywords:** energy harvesting, distributed antenna system, energy efficiency

## Abstract

This paper studies a distributed antenna system (DAS) network with radio frequency (RF) energy harvesting (EH) technology where the distributed antenna ports (DAPs) transmit energy and information to multiple users simultaneously. The time division multiple access (TDMA) protocol is adopted, so for each time slot is allowed to receive information, while the rest of the users harvest energy. In order to maximize the system energy efficiency (EE), subject to the EH requirements and data rate requirements of the users, the transmission time and power assignment are jointly optimized. In order to deal with this non-convex problem, based on Dinkelbach theory and the block-coordinate descent (BCD) scheme, an efficient algorithm is designed to obtain the global optimal solution. Then, simulation results are presented to show that the proposed method achieves much higher system EE compared with benchmark methods. With the increase of the user’s minimum information rate, the system EE decreases rapidly.

## 1. Introduction

Recently, with the rapid growth of the number of users and data traffic of wireless networks, greater coverage and higher information capacity are required to better meet users’ demands. At the same time, many energy-limited wireless devices including Internet of Things (IoT) [1,2,3] devices are expected to be deployed with limited battery capacity, which require being charged frequently.

For the sake of improving the information delivery performance, the distributed antenna system (DAS) is recommended to be employed. Unlike traditional cellular systems, in DAS, the functionality of the base station (BS) is replaced by a central processor (CP) (which may be a mobile edge computing (MEC) device) and a group of distributed antenna ports (DAPs), which extend the network coverage, enhance system capacity, and also improve the service quality of users [4,5,6]. In order to reduce the cost of battery charge and replacement, radio frequency (RF) based wireless energy transfer has been widely regarded as a promising solution, which can be employed to charge the devices wirelessly. However, RF signal transfer suffers from large-scale fading due to the path loss effect [7]. With the assistance of DAS, the RF energy transfer distance can be reduced, and therefore, the RF energy transmission efficiency is greatly enhanced.

In order to inherit the benefits of DAS and RF wireless power transfer, DAS with RF energy harvesting (EH) [8,9,10] have received increasing attention. In [11], the information transmission efficiency of simultaneous wireless information and power transfer (SWIPT)-enabled [12,13,14,15] DAS was maximized. In [16], the system spectral efficiency of DAS with wireless power transmission (WPT) was evaluated. In [17], the authors maximized the network-centric EE and user-centric EE in DAS with WPT. In [18], the authors maximized the EE in DAS with SWIPT [19,20,21], where the power splitting (PS) receiver architecture was employed to fulfill EH and information decoding. In [22], the system goal was to maximize secrecy EE by jointly optimizing the confidential signal’s power, the artificial noise power, and the power-splitting ratio. In [23], the goal was to design a beamforming technique maximizing the secrecy EE, where an efficient iterative algorithm was presented.

This paper also studies the EE in DAS with RF EH. Some differences of our work compared with existing works are outlined as follows:

- Most existing works aimed to maximize the WIT or spectrum efficiency (SE), see, e.g., [11,16,24,25]. With the development of wireless networks, EE becomes more and more important. Therefore, this paper aims to maximize the system EE, to achieve the green communication system design.
- Although some existing works investigated the EE for DAS with RF EH, only the constraint of power control was considered, where the user’s information rate requirement was not involved; see, e.g., [17,18], while in our work, the system EE is maximized with the user’s minimal information rate requirement, which is much closer to the users’ demands.
- Different from the most similar existing works [22,23] where the linear EH model was used, in this work, the nonlinear EH model is adopted, as it was reported that the nonlinear EH one was obtained with real measurement data [26,27]. Thereby, our work may be much closer to the practical situation.

The contributions of this paper are listed as follows.

First, an optimization problem is established to explore the maximum system EE, by jointly optimizing the time assignment and power assignment, subject to the constraints of the minimum achievable information rate requirement, the minimum EH requirement, and the maximum available power.

Second, because of the non-convexity of the fractional objective function of the formulated EE maximization optimization problem, the problem cannot be directly solved using traditional methods. Therefore, a two-layer solution algorithm is designed to seek the optimal solution in an iterative manner. At first, the Dinkelbach theory is applied to handle the problem with a fractional objective function in the outer layer. Then, the joint optimal transmission time and power allocation are found by designing a BCD based algorithm in the inner layer.

Third, simulation results are provided, which show that our proposed method is superior to the benchmark schemes. In particular, our proposed method achieves much higher system EE. Moreover, with the growth of the user’s information rate requirements, the system EE decreases gradually, but its decreasing rate increases.

The remaining sections of this paper are organized as follows. In Section 2, the system model is given, which proposes the DAS structure. Section 3 formulates the EE optimization problem and presents algorithms for the problem to solve the maximal EE. Simulation results are provided to discuss the system performance in Section 4, and finally, the paper is concluded in Section 5.

## 2. System Model

**Figure 1:**
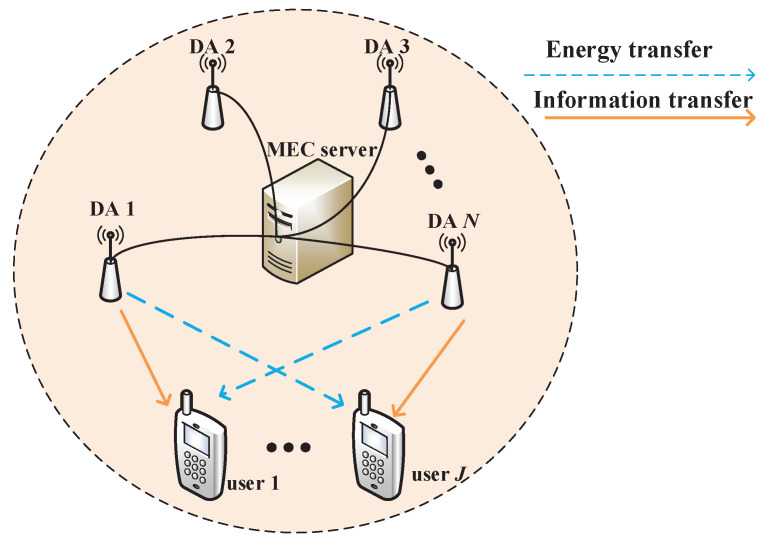
System model of DAS.

A network model as shown in Figure 1 is studied, which is composed of a hybrid access point (HAP), *N* independently powered DAPs, and *J* users. A single antenna is equipped at both DAPs and users. All users desire to get information, as well as energy from the HAP via the RF signals emitted by the DAPs. In order to avoid the information transmission interference among multiple users, TDMA mode is employed in the downlink transmission, where the given time frame with a period of *T* is thus divided into *J* slots. Without loss of generality, *T* is normalized to be one in the sequel. User *j* is only served in the *j*-th time slot with an interval of $\tau_{j}$.

The block fading model is employed to describe the channel. Thus, in each time frame, it is assumed that the channel coefficient is unchanged, and it is changed from one time frame to the next independently. The channel power gain of the link from the *n*-th antenna to the *j*-th user is modeled by (a real number):

(1) $$\begin{array}{c} \;h_{n,j}=ad_{n,j}^{-\phi }\rho_{n,j}^{2},\forall n,j \end{array}$$

where *a* is the path loss with a distance of 1 m and *d* is the distance between DAP *n* and user *j*. ϕ denotes the path loss exponent, and $\rho_{n,j}$ follows an independent and identical distribution (i.i.d.) with unit variance of one and zero mean. The signal received at user *j* from *N* DAPs is expressed by:

(2) $$\begin{array}{c} \;y_{j}=g_{j}^{T}x_{j}+z_{j} \end{array}$$

where $g_{j}={\left[\sqrt{h_{1,j}},\cdots ,\sqrt{h_{N,j}}\right]}^{T}$ (a complex number) represents the vector of the channel coefficients between the *N* DAPs and user *j*, and $x_{j}={\left[x_{1,j},\cdots ,x_{N,j}\right]}^{T}$ represents the user *j*’s transmitted signal vector. $z_{j}$ denotes the additive white Gaussian noise (AWGN). Let $C_{j}=E\left[x_{j}x_{j}^{†}\right]$ be the covariance of the input, following a Gaussian distribution, where ${\left(\cdot \right)}^{†}$ represents the vector’s conjugate transpose. Then, the achievable information rate at user *j* is expressed by:

(3) $$R_{j}=\tau_{j}\log\left(1+\frac{1}{\sigma^{2}}g_{j}^{T}Q_{j}{\left(g_{j}^{†}\right)}^{T}\right).$$

The DAPs are independently distributed and powered throughout the area, so the DAPs do not require joint signal processing and information coding. As a result, the signals transmitted by the *N* DAPs are independent of each other, and therefore, the input covariance matrix is denoted by $C_{j}=\mathrm{diag}\left\{p_{1,j},\cdots ,p_{N,j}\right\}$, where $p_{n,j}$ is the transmit power from DAP *n* to user *j*. Thereby, the achievable information rate at user *j* can be given by:

(4) $$\begin{array}{c} \;R_{j}=\tau_{j}\log\left(1+\frac{\sum_{n=1}^{N}{\left|g_{n,j}\right|}^{2}p_{n,j}}{\sigma^{2}}\right)=\tau_{j}\log\left(1+\frac{\sum_{n=1}^{N}h_{n,j}p_{n,j}}{\sigma^{2}}\right). \end{array}$$

It is noted that $R_{j}$ in (4) presents an achievable rate lower bound by using the maximum ratio transmission (MRT) technique. It is also worth noting that (4) indicates the DAP selection issue, which means that, if DAP *n* is chosen to transmit information to user *j*, $p_{n,j}$ is positive. Otherwise, it is zero.

All users have the EH modules installed. When user *j* is scheduled, it is allowed to decode information from the RF signals. At the same time, the rest of the users $\forall j^{\prime }\neq j$ can harvest energy from the same RF signals. In order to be closer to practice, the piecewise nonlinear EH model [28] is employed. The nonlinear EH model is given by $f\left(x\right)=\left\{\begin{array}{c} \eta x,x<P_{sat}\\ \eta P_{0},x>P_{sat} \end{array}\right.$. At the same time, the mismatch caused cannot be ignored in the system design. Therefore, this paper adopts the nonlinear EH model. Specifically, the energy that can be harvested by user *j* is given by:

(5) $$E_{j}=\left\{\begin{array}{cc} \zeta \sum_{n=1}^{N}h_{n,j}\sum_{j^{\prime }\neq j}^{J}\tau_{j^{\prime }}p_{n,j^{\prime }},\; & \sum_{n=1}^{N}h_{n,j}\sum_{j^{\prime }\neq j}^{J}\tau_{j^{\prime }}p_{n,j^{\prime }}\leq P_{0}\\ \zeta P_{0}, & \sum_{n=1}^{N}h_{n,j}\sum_{j^{\prime }\neq j}^{J}\tau_{j^{\prime }}p_{n,j^{\prime }}\geq P_{0} \end{array}\right.$$

where $P_{0}$ denotes the power threshold received by the users. In (5), $\sum_{j^{{}^{\prime }}\neq j}^{J}\tau_{j^{{}^{\prime }}}p_{n,j^{{}^{\prime }}}$ is the total received energy associated with the signals transmitted to other users. ζ is the energy conversion efficiency, with ζ∈(0,1).

Let $p_{j}^{c}$ be the power consumption of the circuit at user *j*. Then, the energy consumption associated with user *j* can be given by:

(6) $$\begin{array}{c} \;\tau_{j}\left(\sum_{n=1}^{N}p_{n,j}+p_{j}^{c}\right) \end{array}$$

Thus, the total energy consumed by the network can be given by:

(7) $$\begin{array}{c} \;P_{total=}\sum_{j=1}^{J}\tau_{j}\left(\sum_{n=1}^{N}p_{n,j}+p_{j}^{c}\right) \end{array}$$

which is composed of the total transmit power consumed by all DAPs and the total energy consumed by all users.

Then, the system EE of the DAS network can be given by:

(8) $$\begin{array}{c} \;\eta =\frac{\sum_{j=1}^{J}\omega_{j}R_{j}}{P_{total}} \end{array}$$

where $\omega_{j}$ is the non-negative weight associated with user *j*’s EE, which is a constant.

## 3. Problem Formulation and Solution

### 3.1. Problem Formulation

The goal is to maximize the network EE. We assume that the global channel state information (CSI) is known by the MEC server. Denote $p=\{p_{n,j}\}$ and $\tau =\{\tau_{j}\}$. The corresponding optimization problem is mathematically given by:

(P1):maxτ,pη(9)s.t.Ej≥Ej¯,∀j,(10)0≤τj≤1,∀j,(11)0≤pn,j≤Pn¯,∀n,j,(12)∑j=1Jτj≤1,(13)Rj≥Rj,min,∀j,

where $\bar{E_{j}}$ in (9) represents the amount of energy that users need to harvest and $\bar{P_{n}}$ in (11) represents the maximal available power of each DA port. (12) means that the sum of the time slots assigned to the users cannot be larger than *T*. In other words, all users must be scheduled within *T*. (13) indicates that each user’s achievable rate requirement must be satisfied. Problem P1 is non-convex because of its objective function. Thus, we design an efficient solution method in the following subsection.

### 3.2. Problem Analysis

Let q* be the optimal value to problem P1. Then, we have that:

(14) $$\begin{array}{c} q*=\max_{(p,\tau )\in F}\frac{\sum_{j=1}^{J}\omega_{j}R_{j}}{P_{total}} \end{array}$$

where F denotes the feasible solution set to problem P1, which satisfies Constraints (9)–(13). According to [29], problem P1 can be transformed into an equivalent linear form with *q* as follows.

(15) $$\begin{array}{ccc} (P2): & \max_{\left((p,\tau )\in F\right)} & =\sum_{j=1}^{J}\omega_{j}R_{j}-qP_{total}\\ & s.t. & \left(9)(10)(11)(12)(13\right) \end{array}$$

where *q* denotes a feasible solution to problem P1, and problem P1 is equivalently transformed to problem P2 if the following condition, i.e.,

(16) $$\begin{array}{c} T\left(q*\right)=\;\max_{\left((p,\tau )\in F\right)}\;\sum_{j=1}^{J}\omega_{j}R_{j}-qP_{total}=0 \end{array}$$

Therefore, solving problem P1 can be replaced by solving problem P2 instead. Here, the Dinkelbach method [30] is applied to find the optimal q*. In particular, our designed algorithm consists of a two layer framework, and for a given *q*, problem P2 is solved in the inner layer of our presented method. Then, *q* is updated in the outer layer in terms of (13) until the obtained solution satisfies the condition (15).

To effectively deal with problem P2, we first introduce a set of slack variables with $e=\{e_{n,j}\}$. Then, problem P2 can be transformed as:

(17) $$\begin{array}{cccc} \left(P3\right): & \max_{\tau ,e} & = & \sum_{j=1}^{J}\omega_{j}R_{j}-qP_{total}\\ & \;s.t. & & \left(9)(10)(11)(12)(13\right) \end{array}$$

Once the optimal variables (e*,τ*) are obtained by solving problem (P3), one can derive the optimal $p_{{}_{n,j}}^{*}$ by:

(18) pn,j*=en,j*en,j*τj*τj*ifτj*>00ifτj*<0

Moreover, problem P3 is convex, so the Lagrangian dual method is applied to solve it. For a given *q*, the Lagrange function of the problem P3 is:

(19) $$\begin{array}{cc} L\left(e,\tau ,\mu ,\nu ,\lambda ,\delta \right)= & \sum_{j=1}^{J}\omega_{j}\tau_{j}\ln\left(1+\frac{\sum_{n=1}^{N}h_{n,j}e_{n,j}}{\sigma^{2}\tau_{j}}\right)-q\sum_{j=1}^{J}\sum_{n=1}^{N}e_{n,j}-q\sum_{j=1}^{J}\tau_{j}p_{j}^{c}\\ & +\sum_{j=1}^{J}\mu_{j}E_{j}+\lambda \left(1-\sum_{j=1}^{J}\tau_{j}\right)+\sum_{j=1}^{J}\sum_{n=1}^{N}\nu_{n,k}\left(\bar{P_{n}}\tau_{j}-e_{n,j}\right)+\delta_{j}\left(R_{j}-R_{\min}\right) \end{array}$$

where μ,ν,λ,δ are the Lagrangian multipliers respectively related to Constrains (9)–(13). As a result, the Lagrange dual function g(μ,ν,λ,δ) can be given by:

(20) f(μ,ν,λ,δ)=max{0≤τj≤1}{en,j≥0}L(e,τ,μ,ν,λ,δ)

and its dual problem is:

(21) $$\begin{array}{c} \min_{\{\mu ,\nu ,\lambda ,\delta \}\geq 0}\;\;\;f\left(\mu ,\nu ,\lambda ,\delta \right) \end{array}$$

The optimal Lagrangian variables (μ,ν,λ,δ) can be found by solving the problem in (20), where the BCD method [17] can be used, with one of τ and *e* being fixed to optimize the other. There are two cases of $\tau_{j}*$ for a given *e*. First, when $\sum_{n=1}^{N}e_{n,j}=0$, i.e., $e_{n,j}=0$ for all *n*, no energy in this case is transmitted to user *j*, so $\tau_{j}*=0$. When $\sum_{n=1}^{N}e_{n,j}>0$ and ∂2L∂2L∂τj2∂τj2<0, this indicates that the Lagrange functions are concave. If ∂L∂L∂τj∂τj=0, $\tau_{j}*$ can be obtained by:

(22) $$\tau_{j}*=\left\{\begin{array}{cc} \frac{\sum_{n=1}^{N}h_{n,j}e_{n,j}}{\sigma^{2}\left(\exp\left\{\omega \left(-\exp\left\{\frac{A_{j}}{\omega_{j}+\delta }-1\right\}\right)+1-\frac{A_{j}}{\omega_{j}+\delta }\right\}-1\right)} & A_{j}\leq 0,\\ 0 & \mathrm{otherwise}, \end{array}\right.$$

where $A_{j}=\sum_{n=1}^{N}P_{n}\nu_{n,j}-qp_{j}^{c}-\lambda$ and $0\leq \tau_{j}\leq 1$.

With the obtained τ*, the BCD method is used to optimize *e*; that is, with a fixed τ*, the optimized $e_{n,j}$ is found. It is found that the value of ∂L∂L∂en,j∂en,j decreases with the increase of $e_{n,j}$, so *e* can be uniquely determined with ∂L∂L∂en,j∂en,j=0 ($e_{n,j}\geq 0$), i.e.,

(23) $$\begin{array}{c} e_{n,j}*=-\frac{\left(\omega_{j}+\delta \right)\tau_{j}}{B_{n,j}}-\frac{\tau_{j}\sigma^{2}+\sum_{i\neq n}^{N}h_{i,j}e_{i,j}}{h_{n,j}} \end{array}$$

where $B_{n,j}=-q+\zeta \sum_{j^{\prime }\neq j}^{J}\mu_{j^{\prime }}h_{n,j^{\prime }}-\upsilon_{n,j}$. Since the Lagrangian function L is concave w.r.t. the variable (e,τ), the convergence of the BCD method [31] can be ensured.

With the above operations, the optimal solution to Problem (20) is obtained through two layer BCD loops, where in the outer layer, *e* and τ are optimized alternately, and in the inner layer, $e_{n,j}$ is alternatively optimized by fixing other $e_{i,j}$, with a given τ. When *L* does not grow any longer, the iteration stops.

Next, the ellipsoid method [32] is used to obtain the optimal value to the Lagrangian multipliers, and the sub-gradients [30] are used to update as follows,

(24) $$\begin{array}{c} \left\{\begin{array}{c} \Delta \delta_{j}=\tau_{{}_{j}}^{*}\ln\left(1+\frac{\sum_{n=1}^{N}h_{n,j}e_{{}_{n,j}}^{*}}{\sigma^{2}\tau_{{}_{j}}^{*}}\right)-R_{\min}.\forall j,\\ \Delta \lambda =1-\sum_{n=1}^{N}\tau_{{}_{j}}^{*},\\ \Delta \nu_{n,j}=\bar{P_{n}}\tau_{{}_{j}}^{*}-e_{{}_{n,j}}^{*},\forall n,j,\\ \Delta \mu_{j}=\zeta \sum_{n=1}^{N}h_{n,j}\sum_{j^{{}^{\prime }}\neq j}^{J}e_{n,j^{{}^{\prime }}}-\bar{E_{j}},\forall j. \end{array}\right. \end{array}$$

Finally, after completing the above steps to solve the dual function, update *q* until *q* converges to a certain accuracy, also outputting the optimal solution to problem P1. Algorithm 1 is as follows.

The complexity of the BCD method is $O(NJ^{2})$, and the complexity of the ellipsoid method is $O({\left(JN+J+1\right)}^{2})$. Thus, the total complexity of Algorithm 1 is $O(NJ^{2}{\left(JN+J+1\right)}^{2}I)$, where I is the number of iterations for updating *q*. In Section 4, our simulation results will show that the proposed method achieves much higher system EE compared with the benchmark methods.
**Algorithm 1:** The presented solution approach for solving problem P1.1 **Initialize***q*2 **While**
T(q*)≤ε, **do**3  Initialize {μ,ν,λ,δ}4  **While**
{μ,ν,λ,δ}≤ε, **do**5   Initialize *e* and τ6   **While**
*L* stops the improvement, **do**7    Compute the $\tau_{j}$ that maximizes *L*. 8    Compute *e* with fixed τ. 9  Update the Lagrangian multipliers of {μ,ν,λ,δ} with the ellipsoid method via subgradients. 10 Update *q*11 **Obtain**
p*. 

## 4. Simulations and Discussion

This section performs some simulations to evaluate the system performance of the proposed scheme. For clarity, Table 1 summarizes the simulation parameters. In the simulations, *N* DAPs are evenly distributed in a 30 m × 30 m square area. All users are distributed within the area randomly. A baseline scheme on maximizing EE with a fixed time allocation (referred as to “FT” in the figures) is also simulated for comparison. Specifically, in the FT scheme, the information transmission time for each user is equally assigned with τj=11JJ,∀j, and the complexity of FT scheme is $O(NJ^{4})$. Moreover, in the figures, our presented scheme is marked by “OPT” for simplicity.

**Figure 2:**
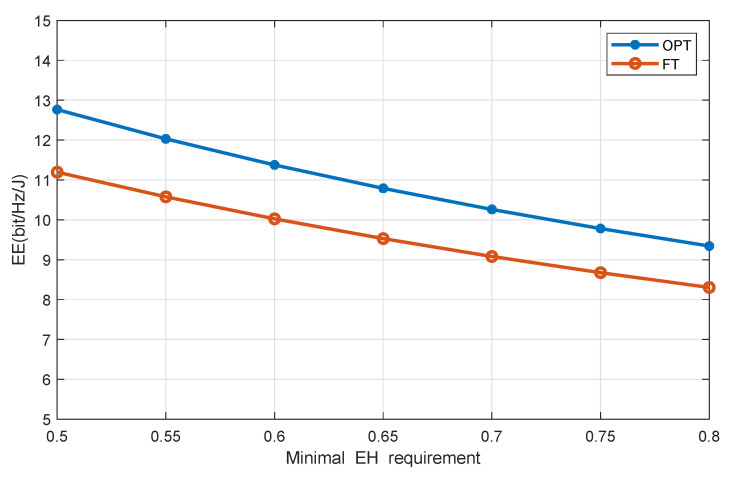
System EE versus users’ EH requirement. FT, fixed time allocation.

Figure 2 discusses the effect of the minimum EH requirement on system EE where the number of users is four and that of the distributed antenna ports is seven. It is observed that our presented scheme achieves higher system EE compared with the benchmark one. Moreover, when the demand for the minimum EH requirement increases, the EE decreases. There may be two reasons. First, with the higher minimum EH requirement, more time is required by the users to harvest energy to meet their growing EH demand, which may reduce the time for the users to decode the information and, then, reduces the throughput. Second, as the minimum EH requirement increases, the DAPs may transmit higher power to allow users to receive more energy, leading to increased energy consumption.

**Figure 3:**
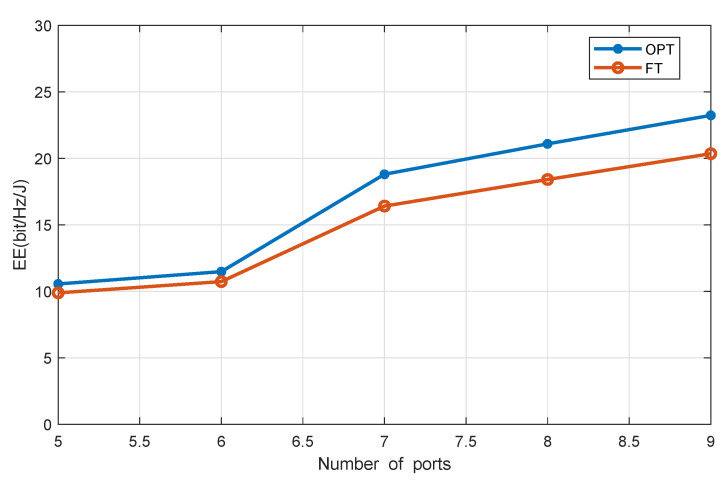
EE versus the number of DAPs.

Figure 3 shows the system EE versus the number of DAPs, with J=4 and the users’ EH requirement being 0.2 mW. Compared with the benchmark scheme, i.e., NC-FT, our proposed scheme achieves higher system EE. Moreover, the more DAPs, the higher system EE is, because with more DAPs, the average access distance between users and DAPs is shortened, which enhances system efficiency.

**Figure 4:**
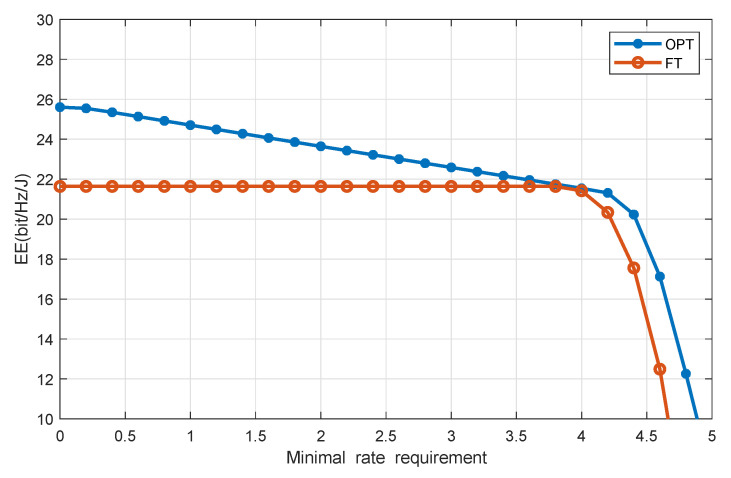
EE versus users’ minimal rate requirement.

Figure 4 plots EE versus the minimum throughput requirement with J=4 and the users’ EH requirement being 0.2 mW. It is seen that EE decreases as the minimum throughput requirement increases, because when the minimum throughput requirement increases, more transmit power is needed by the users, thereby causing a reduction of system EE.

**Figure 5:**
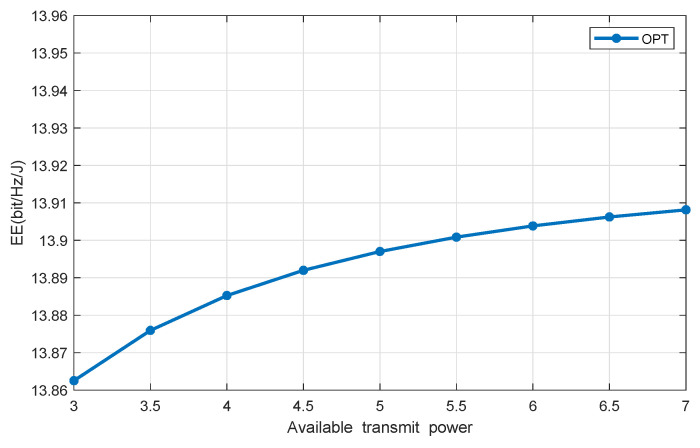
EE vs. available transmit power in OPT.

**Figure 6:**
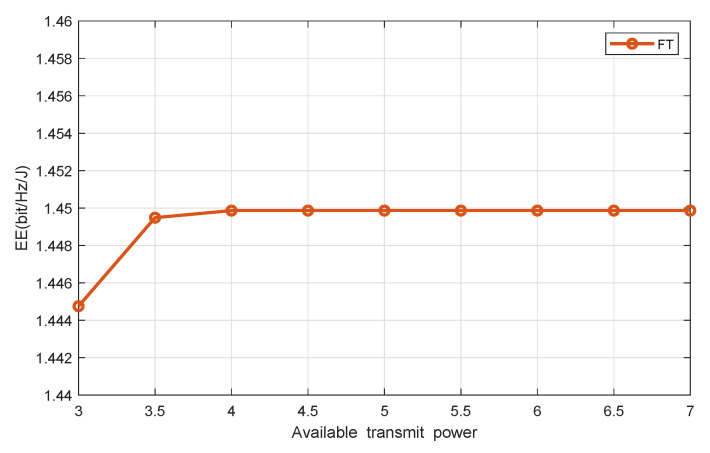
EE vs. available transmit power in FT.

Figure 5 and Figure 6 show the system EE versus the available HAP’s transmit power, with the number of DAPs and users being seven and four, respectively. It shows that our proposed scheme achieves much higher system EE than the benchmark one. With the increment of the available HAP’s transmit power, the achieved system EE of both our proposed scheme and the reference scheme increases. In particular, the EE increases sharply at first and then gradually becomes saturated in a high transmit power region, because when the HAP’s available transmit power is relatively small, the minimal EH requirement is relatively high, which means that the user demands are higher and the network resource is less. Therefore, in this region, by increasing the lunit of transmit power, the system EE is significantly improved. When the available transmit power is relatively large, network resources are abundant, which can easily meet the minimum demand for EH. In this case, the available transmit power may not be used up. Therefore, increasing $\bar{P_{n}}$ will not affect EE any longer.

## 5. Conclusions

This paper studied a DAS with RF EH, where the DAPs transmit energy and information to multiple users simultaneously. TDMA mode was employed, so each time slot was allowed to receive information, while the rest of the users have harvested energy. In order to explore the system EE, subject to the EH requirements and data rate requirements of the users, the power allocation and time assignment were optimized jointly. To handle this non-convex problem, we designed an efficient algorithm on the basis of the Dinkelbach theory to find the optimal solution. Simulation results showed that our proposed method achieved much higher system EE than other benchmark methods. Moreover, with the growth of the user’s requirement for the minimum information rate, the system EE decreases, and the decreasing rate also increases.

## Figures and Tables

**Table 1:** Simulation parameter setting.

Parameters	Notation	Value
Noise power	$\sigma^{2}$	$10^{-10}$ W
Path loss at a reference distance of 1m	*a*	$10^{-1}$
Energy conversion efficiency	ζ	0.6
Length of the square	*l*	30 m
Path loss exponent	ϕ	2
Circuit power consumption	$p_{k}^{c}$	0.5 W
Weight of users	$\omega_{k}$	1

## Abbreviations

The following abbreviations are used in this manuscript:

DAS	distributed antenna system
DAPs	distributed antenna ports
RF	radio frequency
TDMA	time division multiple access
EE	energy efficiency
IoT	Internet of Things
EH	energy harvesting
WPT	wireless power transmission
SWIPT	simultaneous wireless information and power transfer
MEC	mobile edge computing
HAP	hybrid access point
BCD	block-coordinate descent
MRT	maximum ratio transmission
AWGN	additive white Gaussian noise
